# Cortical Shape and Curvedness Analysis of Structural Deficits in Remitting and Non-Remitting Depression

**DOI:** 10.1371/journal.pone.0068625

**Published:** 2013-07-16

**Authors:** Yuan-Lin Liao, Po-Shan Wang, Chia-Feng Lu, Chih-I Hung, Cheng-Ta Li, Ching-Po Lin, Jen-Chuen Hsieh, Tung-Ping Su, Yu-Te Wu

**Affiliations:** 1 Department of Biomedical Imaging and Radiological Sciences, National Yang-Ming University, Taipei, Taiwan; 2 Brain Research Center, National Yang-Ming University, Taipei, Taiwan; 3 Institute of Brain Science, National Yang-Ming University, Taipei, Taiwan; 4 The Neurological Institute, Taipei Municipal Gan-Dau Hospital, Taipei, Taiwan; 5 Department of Neurology, National Yang-Ming University School of Medicine, Taipei, Taiwan; 6 Department of Neurology, Neurological Institute, Taipei Veterans General Hospital, Taipei, Taiwan; 7 Department of Physical Therapy and Assistive Technology, National Yang-Ming University, Taipei, Taiwan; 8 Department of Education and Research, Taipei City Hospital, Taipei, Taiwan; 9 Department of Psychiatry, Taipei Veterans General Hospital, Taipei, Taiwan; 10 Faculty of Medicine, National Yang-Ming University, Taipei, Taiwan; 11 Institute of Neuroscience, National Yang-Ming University, Taipei, Taiwan; 12 Integrated Brain Research Laboratory, Department of Medical Research and Education, Taipei Veterans General Hospital, Taipei, Taiwan; 13 Center for Neuropsychiatric Research, National Health and Research Institute, Taipei, Taiwan; I2MC INSERM UMR U1048, France

## Abstract

In morphometric neuroimaging studies, the relationship between brain structural changes and the antidepressant treatment response in patients with major depressive disorder has been explored to search depression-trait biomarkers. Although patients were treated with serotonin-related drugs, whether the same treatment resulted in remission and non-remission in depressed patients is currently under investigation. We recruited 25 depressed patients and 25 healthy controls and acquired volumetric magnetic resonance imaging of each participant. We used the shape index and curvedness to classify cortical shapes and quantify shape complexities, respectively, in studying the pharmacological effect on brain morphology. The results showed that different regions of structural abnormalities emerged between remitting and non-remitting patients when contrasted with healthy controls. In addition to comparing structural metrics in each cortical parcellation, similar to the traditional voxel-based morphometric method, we highlighted the importance of structural integrity along the serotonin pathway in response to medication treatment. We discovered that disrupted serotonin-related cortical regions might cause non-remission to antidepressant treatment from a pharmacological perspective. The anomalous areas manifested in non-remitting patients were mainly in the frontolimbic areas, which can be used to differentiate remitting from non-remitting participants before medication treatment. Because non-remission is the failure to respond to treatment with serotonin-related drugs, our method may help clinicians choose appropriate medications for non-remitting patients.

## Introduction

Major depressive disorder (MDD), or unipolar depression, is the single most important contributor to the total burden of neuropsychiatric disorders in the European Union [1]. MDD patients experience one or more major depressive episodes–a minimum of 2 weeks of the following possible symptoms: changes in weight, sleep, and appetite; psychomotor agitation or retardation; loss of energy; difficulty with thinking, concentration, or decision making; feelings of worthlessness or guilt; and/or suicidal ideation. Pharmacotherapy is one choice for effective treatment of MDD patients to achieve remission and recovery. In the 17-item Hamilton Depression Rating Scale (HAMD-17) [2], remission requires a score of 7 or less [3]. However, not all patients who undergo treatment respond well, and up to one-third fail to achieve remission, despite multiple drug trials [4]. A patient might also relapse from remission and recovery to enter a new episode [3]. In a recent large-scale cohort study using a nationwide database in Taiwan, patients with a poor antidepressant response exhibited a higher rate of change to bipolar disorder in a subsequent diagnosis [5].

Although newly developed antidepressants have been introduced and used in MDD treatment, selective serotonin reuptake inhibitors (SSRIs) remain the most widely prescribed drugs. The serotonergic system is the main target of SSRIs, which increase serotonin levels by blocking the serotonin transporter. The distributions of serotonin, a specific serotonin pathway, originate in the midline of the brain stem, the raphe nuclei, and spread upwardly to the substantia nigra, the remaining basal ganglia, the thalamus, hypothalamus, cortex, amygdala, and hippocampi. SSRI treatments have shown the efficacy of improving cognitive function in MDD patients [6]. Serotonin-transporter binding has become a possible predictor of treatment response in depressed patients [7]. Later studies have also recognized the increasing importance of norepinephrine. Antidepressant drugs that combine serotonergic and noradrenergic action mechanisms were more effective than the sole use of SSRI in treating MDD [8]. The locus ceruleus serves as the major norepinephrine source, and the axons project in the median forebrain bundle where they distribute to the hypothalamus, thalamus, basal ganglia, amygdale, hippocampus, and entire neocortex. Serotonin and norepinephrine pathways share the same feature in that they mainly distribute over the medial cortex.

Dysfunctional neurocircuitry of cognitive and emotional processing has been reported in MDD patients [9]. Researchers have used neuroimaging techniques to detect the brain-functional disturbance [10] and to locate cortical structural deficits [11] in the related cortical areas. Various anatomical studies have emerged in the literature investigating the alterations in cortical volume [12], folding [13], and thickness [14] through structural magnetic resonance (MR) images. Cortical shape analysis can be performed by measuring cortical complexity, which relates to the frequency of folding and the degree of gyral convolution (i.e., gyrification) on the cortex. Several studies have shown structural changes in cortical complexity in patients with affective disorder. Penttilä et al. found that intermediate-onset bipolar disorder (BD) patients had a significantly reduced local sulcal index in the right dorsolateral prefrontal cortex versus both early-onset BD patients and healthy participants, and lower global sulcal indices in both hemispheres versus healthy participatns [15]. Zhang et al. found a reduced local gyrification index in the bilateral mid-posterior cingulate, insula, and orbital frontal cortices, the left anterior cingulate cortex, and the right temporal operculum [13]. In addition to the commonly used metrics (eg, the gyrification index [16] and fractal dimension [17]), which belong to regional-level descriptors quantifying the folding of a specific cortical area, the use of voxel-level (vertex-level) metrics (eg, mean and Gaussian curvatures [18]) is also useful in cortical development and disease progress. The combined shape index (SI) and curvedness (CVD) proposed by Awate et al. [19] is a novel approach to investigate folding patterns. In a recent study, Hu et al. used SI to identify what cortical shape each voxel belongs to (i.e. sulcal pit, sulcal saddle, gyral saddle, or gyral node), and CVD to quantify how it deviates from a flat plane [20]. For each classified region, the mean CVD values were used to analyze fetal brain development. This approach provides additional insights into brain morphology in unraveling aberrant changes.

Numerous structural neuroimaging studies involving both remitting and non-remitting MDD participants have focused on antidepressant-treatment response related to brain volumetric changes to search depression-trait biomarkers [21–23], and to investigate structural-deficit differences [24]. Liu et al. used multivariate pattern analysis on structural MR scans to classify MDD patients with different therapeutic responses and healthy controls [25]. Li et al. investigated structural abnormalities and cognitive deficits, but ignored the possible causal relationships between antidepressant drugs and volumetric alterations [26]. Salvadore et al. [27] and Caetano et al. [28] recruited unmedicated depressed patients to rule out the neurotrophic effects of antidepressant drug exposure and studied only depressive-state characteristics. Whether the same treatment resulted in different effects on depressed patients is currently under investigation. Little consensus has been reached for why the proportions of non-remitting patients was so high. Treatment-failure factors have previously been explained by incorrect diagnoses, inadequate dosage, treatment duration, or psychiatric and medical comorbidities [29], [30]. However, recent studies have shown differences between the brains of remitting and non-remitting patients. We used the SI combined with CVD to analyze the cortical shape complexity of remitting and non-remitting patients to determine whether the difference between the 2 groups may explain their antidepressant response.

## Materials and Methods

### Participants

The MDD patients were recruited from outpatients of the Psychiatric Department of Taipei Veterans General Hospital and diagnosed using the Diagnostic and Statistical Manual of Mental Disorders, fourth edition, text revision (DSM-IV-TR). After screening, participants received open-label antidepressant treatment (SSRIs, SNRIs, or bupropion) for the next 6 weeks. Of the 25 adults with recurrent MDD, 13 achieved remission (male/female = 3/10, age mean ± S.D. = 37±10 y, age range = 24–56 y, HAMD-17 scores mean ± S.D. = 3.077±2.139) and 12 were non-remitters (male/female = 6/6, age mean ± S.D. = 37±10 y, age range = 23–48 y, HAMD-17 scores mean ± S.D. = 15.667±6.624). We used the remission criterion of a participant score of 7 or less in the 17-item Hamilton Depression Rating Scale. In addition, 25 age- and sex-matched healthy controls (male/female = 10/15, age mean ± S.D. = 37±12 y, age range = 21–57 y) were recruited and evaluated by psychiatrists using the Mini International Neuropsychiatric Interview (M.I.N.I.) to exclude the possible morbidity of major psychiatric illness. The demographic data of all subjects and clinical parameters of MDD patients are listed in Table 1. The study was performed in accordance with the Declaration of Helsinki and was approved by the Ethics Review Committee of Taipei Veterans General Hospital. Written informed consent approved by the institutional review board from all participants was obtained.

**Table 1:** Demographic information and clinical parameters.

Variable	Non-remitting MDD (*n* = 12)	Remitting MDD (*n* = 13)	HC (*n* = 25)
Age^a^ (years)	37±10	37±10	37±12
Gender (*n* = male/female)	6/6	3/10	10/15
Age of onset^a^ (years)	28.000±9.789	25.923±8.301	−
Duration of illness^a^ (years)	9.083±7.267	10.846±8.896	−
Past depressive episodes^a^ (times)	5.667±3.916	3.692±2.898	−
HAMD-17 scores^a^	15.667±6.624	3.077±2.139	−
YMRS scores^a^	1.250±2.050	0.385±0.650	−
*Medication*			
SSRI (*n*)	4	5	−
SNRI (*n*)	5	4	−
SARI (*n*)	2	0	−
NDRI (*n*)	6	2	−
TCA (*n*)	1	0	−

MDD: major depressive disorder; HC: healthy controls; HAMD-17: Hamilton Depression Rating Scale, 17 items; YMRS: Young Mania Rating Scale; SSRI: selective serotonin reuptake inhibitor; SNRI: serotonin norepinephrine reuptake inhibitor; SARI: serotonin antagonist reuptake inhibitor; NDRI: norepinephrine dopamine reuptake inhibitor; TCA: tricyclic antidepressant.

a Continuous variables are expressed as mean±standard deviation (SD).

### Image Acquisition

Structural brain images were acquired using a 1.5 Tesla MR scanner (General Electric, Milwaukee, WI, USA) with a 3D fast-spoiled gradient-recalled (3D-FSPGR) T1 sequence to obtain 124 axial slices with an in-plane resolution of 1.02×1.02 mm^2^. The imaging parameters were TR/TE/TI = 8.54/1.84/400 ms, field-of-view (FOV) = 260 mm, matrix size = 256×256, slice thickness = 1.5 mm, NEX = 1, flip angle = 15°.

### Image Processing

The anisotropic images were resampled into volumes with isotropic voxel dimensions of 1.02×1.02×1.02 mm^3^ to facilitate the computation of cortical complexity. We located the anterior commissure and posterior commissure using the Automatic Registration Toolbox [31] to determine the midsagittal plane, which was then aligned parallel to the YZ volume plane. Skull-stripping and intensity non-uniformity correction were performed using the hybrid watershed algorithm [32] and non-parametric, non-uniform intensity normalization [33], respectively, in Freesurfer (available online at http://surfer.nmr.mgh.harvard.edu/). We classified the brain tissue into gray matter (GM), white matter (WM), and cerebrospinal fluid (CSF) using SPM8 (Statistical Parametric Mapping, version 8; available online at http://www.fil.ion.ucl.ac.uk/spm/). The outer surface of cortex was delineated as the GM/CSF boundary. The extracted outer surface was validated by two experienced neuroradiologists (PS Wang and TP Su) and was manually adjusted if necessary. In the subsequent analysis, we only computed the shape index and curvedness on the outer surface to observe the changes of cortical surface morphology since we hypothesized that the impairment of target of serotonin (serotonin receptors in the GM) caused the therapeutic differences between remitted and non-remitted MDD patients. To exclude the pathological changes in WM irrelevant to our serotonin hypothesis, such as the degeneration or demyelination of axons that may affect the morphology of inner surface (GM/WM surface), the morphological changes of inner surface were not considered. Ninety gross cortical structures on the cerebral gray matter were extracted and labeled based on Anatomical Automatic Labeling (AAL) [34] by the Individual Brain Atlases using Statistical Parametric Mapping (IBASPM) software [35].

### Estimating Cortical Shape Complexity

In our previous study [20], the combined use of SI and CVD showed both the surface type and the magnitude of shape complexity. These two metrics have been used to study cerebral cortical folding on sex differences and neonatal development [19]. Other studies have also emerged to accentuate the significance of classifying cortical shapes into gyri and sulci in the analysis [36–38]. Our group recently proposed using SI in classifying the cortical surface and CVD in measuring the degree of deviation of cortical shapes from a flat plane. The separation between gyral and sulcal shapes effectively provided additional morphological information in fetal brain development [20]. Similarly, we used a binarized volume of interest to compute the shape metrics for each parcellated cortical region (i.e., we used the 100 and 0 intensity to represent the object and background voxels). We adopted an intensity-based estimation method, implemented in the DIPimage toolbox (a MATLAB toolbox; available online at http://www.diplib.org/) to obtain the principal curvatures directly from the volume image without explicitly reconstructing the surface [39]. SI and CVD were then computed from the 2 principal curvature values *k*_1_ and *k*_2_ as follows:
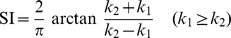


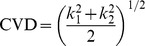



We focused only on the surface voxels of cortical regions. Based on the SI value, each voxel can be classified as gyral nodes (0.5<SI<1), sulcal pits (−1<SI<−0.5), and 2 transitional shapes between gyral nodes and sulcal pits–the gyral saddle (0<SI<0.5) and the sulcal saddle (−0.5<SI<0). We averaged and used CVD values in the same regional category for further statistical analysis and obtained 4 mean CVD values for each of the 90 cortical regions for every participant. To alleviate the effect of brain size differences in the computation of curvedness, we have corrected the CVD by multiplying it with the intra-cranial volume (ICV) ratio, which is defined as follows [19]:
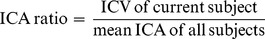



### Statistical Analysis

We performed the ANOVA statistics to examine the group differences in cortical shape complexity for each brain partition. When a group difference was detected (*P*<.05), three possible pairwise *t*-tests (non-remitting MDD patients vs. healthy controls, remitting MDD patients vs. healthy controls, and remitting vs. non-remitting MDD patients) were conducted with Bonferroni correction of *P* values. Statistical significance was set at *P*<.05 with pairwise *t*-tests.

## Results

**Figure 1:**
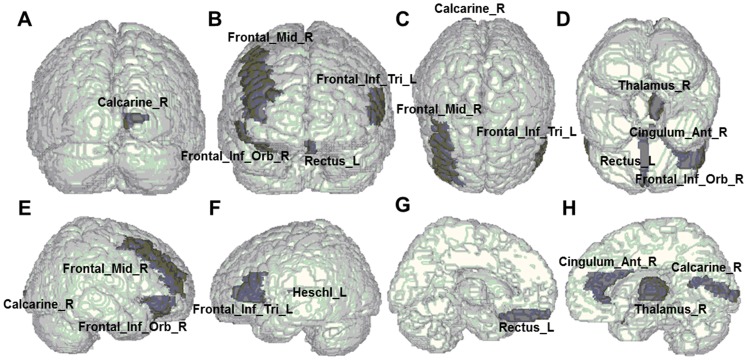
Anatomical regions exhibiting significant differences of cortical shape complexity between non-remitting depressed patients and healthy controls. The curvedness values significantly differed between non-remitting patients and healthy controls in the the right middle frontal gyrus (Frontal_Mid_R), the orbital part of right inferior frontal gyrus (Frontal_Inf_Orb_R), the left gyrus rectus (Rectus_L), and the right calcarine fissure and surrounding cortex (Calcarine_R) in the sucal pits. Significant differences also existed in the gyral saddle parts of the right thalamus (Thalamus_R) and the left Heschl gyrus (Heschl_L) and the gyral nodes of the right anterior cingulate and paracingulate gyri (Cingulum_Ant_R). In the triangular part of left inferior frontal gyrus (Frontal_Inf_Tri_L), we also discovered significant differences in the gyral saddle and sulcal saddle areas, respectively. All areas exhibiting significant differences were shown in (A) posterior, (B) anterior, (C) superior, (D) inferior (E) right-lateral, (F) left-lateral, (G) left-medial, and (H) right-medial views.

**Figure 2:**
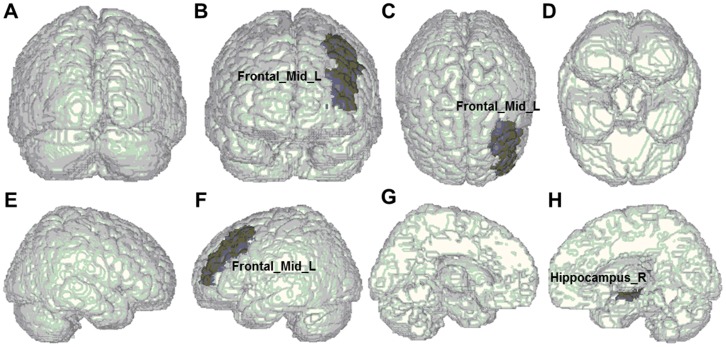
Anatomical regions exhibiting significant differences of cortical shape complexity between remitting depressed patients and healthy controls. In comparing remitting patients with healthy controls, the significant differences of the curvedness values were only in the sulcal pits of the left middle frontal gyrus (Frontal_Mid_L) and the right hippocampus (Hippocampus_R) as shown in (A) posterior, (B) anterior, (C) superior, (D) inferior (E) right-lateral, (F) left-lateral, (G) left-medial, and (H) right-medial views.

**Figure 3:**
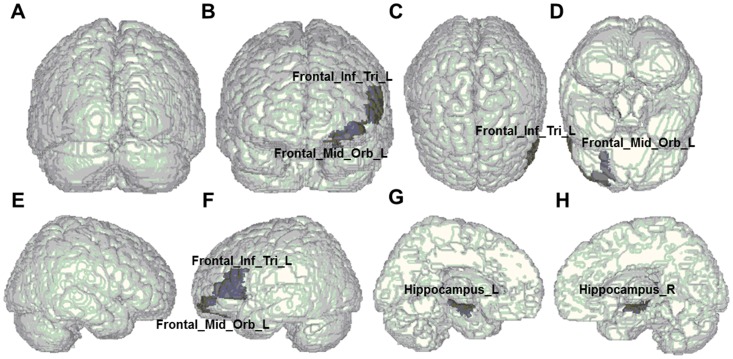
Anatomical regions exhibiting significant differences of cortical shape complexity between remitting and non-remitting depressed patients. The regions showing significant differences were the sulcal pits of the orbital part of left middle frontal gyrus (Frontal_Mid_Orb_L), the gyral saddle areas of the triangular part of left inferior frontal gyrus (Frontal_Inf_Tri_L), the gyral nodes of left hippocampus (Hippocampus_L), and the sulcal pits of right hippocampus (Hippocampus_R) as shown in (A) posterior, (B) anterior, (C) superior, (D) inferior (E) right-lateral, (F) left-lateral, (G) left-medial, and (H) right-medial views.

The CVD values significantly differed between non-remitting MDD patients and healthy controls in the right middle frontal gyrus (*P* = .032), the orbital part of right inferior frontal gyrus (*P* = .025), the left gyrus rectus (*P* = .010), and the right calcarine fissure and surrounding cortex (*P* = .041) in the sucal pits (Table 2). Significant differences also existed in the gyral saddle parts of the right thalamus (*P* = .012) and the left Heschl gyrus (*P* = .029) and the gyral nodes of the right anterior cingulate and paracingulate gyri (*P* = .030). In the triangular part of left inferior frontal gyrus, we also discovered significant differences in the gyral saddle (*P* = .003) and sulcal saddle areas (*P* = .017), respectively. In addition, we found all areas exhibiting significant differences between non-remitting MDD patients and healthy controls are shown in Fig. 1. In comparing remitting MDD patients with healthy controls, the significant differences were only in the sulcal pits of the left middle frontal gyrus (*P* = .049) and the right hippocampus (*P* = .026), shown in Fig. 2. Additionally, we made a comparison between remitting and non-remitting MDD patients. The significantly different regions were the sulcal pits of the orbital part of left middle frontal gyrus (*P* = .044), the gyral saddle areas of the triangular part of left inferior frontal gyrus (*P* = .011), the gyral nodes of left hippocampus (*P* = .050), and the sulcal pits of right hippocampus (*P* = .005) as shown in Fig. 3.

**Table 2:** Comparison of cortical shape complexities estimated by mean curvedness between non-remitting and remitting MDD patients in contrast with healthy controls.

Anatomical region^a^	Type^b^	MDD^c^	HC^c^	adjusted *P*-value
*non-remitting*				
Frontal_Mid_R	Sp	.350 (.006)	.369 (.005)	.032219
Frontal_Inf_Tri_L	Gs	.244 (.006)	.272 (.005)	.002505
Frontal_Inf_Tri_L	Ss	.317 (.005)	.341 (.005)	.017265
Frontal_Inf_Orb_R	Sp	.363 (.007)	.343 (.004)	.025329
Rectus_L	Sp	.287 (.007)	.263 (.004)	.010358
Cingulum_Ant_R	Gn	.258 (.004)	.240 (.004)	.029842
Calcarine_R	Sp	.380 (.013)	.347 (.007)	.041001
Thalamus_R	Gs	.315 (.028)	.240 (.012)	.012373
Heschl_L	Gs	.264 (.016)	.331 (.015)	.028620
*Remitting*				
Frontal_Mid_L	Sp	.353 (.004)	.368 (.003)	.049176
Hippocampus_R	Sp	.281 (.005)	.299 (.004)	.026289

MDD: major depressive disorder; HC: healthy controls.

a Anatomical regions are listed as the labels in the Anatomical Automatic Labeling (AAL) atlas; Frontal_Mid_R: right middle frontal gyrus; Frontal_Inf_Tri_L: left inferior frontal gyrus, triangular part; Frontal_Inf_Orb_R: right inferior frontal gyrus, orbital part; Rectus_L: left gyrus rectus; Cingulum_Ant_R: right anterior cingulate and paracingulate gyri; Calcarine_R: right calcarine fissure and surrounding cortex; Thalamus_R: right thalamus; Heschl_L: left Heschl gyrus; Frontal_Mid_L: left middle frontal gyrus; Hippocampus_R: right hippocampus.

b Ss: sulcal saddle; Gs: gyral saddle; Sp: sulcal pit; Gn: gyral node.

c Curvedness values are given as mean (standard deviation).

## Discussion

We assessed the shape complexity of cortical parcellations using structural MR brain images. Similar procedures proposed in our previous study [20] were followed to classify the cortical shapes and measure the degree of shape complexity using SI and CVD, respectively. We compared these quantified metrics in each parcellated cortical region between patients with remitting and non-remitting MDD when contrasting with healthy controls, and found that the defected areas in non-remitting patients distributed mainly along the serotonin pathway.

In the comparison between remitting patients and healthy controls, we discovered 2 abnormal areas in the left middle frontal gyrus and the right hippocampus in depressed patients, which have also been mentioned in previous studies. Wagner et al found a reduction of gray matter volume in the left middle frontal gyrus [40]. Du et al reported gray matter decreases in the right hippocampus in a voxelwise meta-analysis [41]. Lim et al showed reduced volume of the right hippocampus in drug-naïve patients with late-onset depression [42]. Our results in remitting patients were consistent with previous findings.

Our results indicate that structural deficits in non-remitting patients are located mainly in the serotonin-related cortical regions. Because depressed patients took medications that were mostly SSRI/SNRI-related drugs, we suggest that the defected target organs resulted from the absence of a treatment response in medication-resistant patients and brain dysfunctions. This novel finding has not been previously discovered, and studies have not noted that the structural changes along the neurotransmitter pathway may result from failure to remit.

The abnormal serotonin neurotransmitter system and the alterations of serotonin transporter are important in MDD pathophysiology [43]. Parsey and colleagues studied one serotonin receptor, the 5-HT_1A_ receptor, and discovered that higher 5-HT_1A_ binding is associated with a poorer response to antidepressant treatment [44]. In a later study of the same group, Miller et al found that lower serotonin transporter binding may predict non-remission of MDD [7] and elevated 5-HT_1A_ binding in remitting depressed participants [45]. Accordingly, serotonin played a critical role in the response to medication treatment. Cortical structural abnormalities along the serotonin pathway may be indicators of treatment response. These previous findings are in accordance with our results of serotonin system disturbance in non-remitting patients and concur with our hypothesis that structural deficits along the serotonin pathway may result in non-remission of MDD patients.

Although current anatomical studies mostly use voxel-based morphometry (VBM) to locate brain regions of interest, our findings obtained from comparing cortical shape complexity between depressed patients and healthy controls are similar to volumetric alterations resulting from VBM. We found that the disrupted areas of non-remitting patients are mainly distributed over the fronto-limbic regions. This is consistent with a literature review [46] in which the middle frontal gyrus and anterior cingulate cortex were reported to be potentially predictive regions of treatment response. In discriminating treatment-resistant from treatment-sensitive depression, Liu et al showed the left middle frontal gyrus and the left inferior frontal gyrus to be 2 of the most important gray matter regions [25]. This corresponded to our finding that the significant differences between remitting and non-remitting depressed patients showed in the orbital part of left middle frontal gyrus and the triangular part of left inferior frontal gyrus. Although these papers focused on the comparison between remitting and non-remitting participants, they only presented the relationship between structural and functional deficits, and studies have rarely investigated the connection of antidepressant treatment and structural abnormalities. In contrast, we discovered that the anomalous areas manifested in non-remitting patients distributed mainly along the serotonin pathway. The disruption of their target organs may explain why certain MDD patients did not respond to SSRI/SNRI treatment.

In this study, we classified cortical shapes and quantified shape complexity to facilitate examining the pharmacological effect on brain morphology. We discovered the regional difference of structural abnormalities between remitting and non-remitting MDD patients in contrast with healthy controls. In addition to comparing the structural metrics in each cortical parcellation, similar to the traditional VBM method, we highlighted the importance of structural integrity along the serotonin pathway in response to medication treatment. Although all MDD patients were treated with SSRI and SNRI medications, certain patients remitted, whereas others did not. Our investigation on cortical shape complexity suggests that the disruption of serotonin-related cortical regions may be the cause of non-remission to SSRI/SNRI treatment. The anomalous areas manifested in non-remitting patients were mainly in the frontolimbic areas, and can be used to differentiate remitting from non-remitting patients before medication treatment. Because non-remission is the failure to respond to SSRI/SNRI treatment, our method may help clinicians choose appropriate medications for non-remitting patients.
